# Uterine Fibroids

**Published:** 1903-05-09

**Authors:** 


### UTERINE FIBROIDS.

One of the most important results of the steady advance of modern surgery is the growing tendency in favour of early surgical interference in chronic affections which are amenable to operation. A succession of articles published in the medical journals of late has shown how this principle of early operation is coming more and more into favour. In a clinical lecture given at the School of Medicine for Women and published in the *Clinical Journal*, Dr. Mary Sharlieb discusses the question in its bearing on fibroid disease of the uterus. She calls attention to the fact that fibroid tumours of the uterus are more dangerous than is commonly supposed. With regard to this point there are no general statistics which can be considered in any way reliable guides either as to the frequency of the disease or to the mortality which, directly or indirectly, it brings about. She points out that many patients who have fibroids die of some disease which is really connected with the tumours, but in which the connection is not always recognised. For example, women die of heart degeneration, of pleurisy, or of kidney trouble. Any one of these affections may arise independently of fibroid disease, or may be consecutive to it, but they appear in the death certificate under their obvious headings, and the fibroid, whether the cause or not, receives no mention. The most important question for decision in dealing with patients who are suffering from the affection is when to advise removal of the tumours by operation. In some cases, especially those where complications have supervened, there can be little doubt, in others the *pros* and *cons* should be explained to the patient, and she should decide for herself. Every case has to be dealt with on its own merits rather than on general rules. Thus the proximity of the menopause, the condition of the patient whether married or single, her station in life, must all be considered in individual cases. The most important general indications for operation are hæmorrhage, pain, pressure symptoms, rapid growth, pregnancy and sloughing of the tumour. But in estimating the value of these phenomena individual differences must be taken into account. Some women can withstand repeated and copious loss of blood for a long time without their general health deteriorating, while in other cases marked anæmia may soon appear, and it must be remembered that repeated loss of blood causes anæmia, not merely by depletion, but, when extended over a considerable period, by destroying the patient's power of making blood. Pain is not necessarily an indication. It may be due to a displaced fibroid becoming impacted in the pelvis, and it may be possible to push up the fibroid and prevent a recurrence of the prolapse by the use of a ring pessary. Pressure symptoms, unless due to impaction of a fibroid in the pelvis, nearly always indicate myomectomy. Rapid increase generally means that some degeneration has occurred, such as that which results in the formation of cysts. It is usually associated with pain, often with pressure symptoms, and sometimes with a septic condition. Pregnancy is a grave complication of fibroid disease. The dangers are :—(1) Abortion, which under these circumstances has, Dr. Sharlieb states, a mortality of 36 per cent. (2) Hæmorrhage after delivery. (3) Sepsis after delivery. Bearing in mind that we have no method of treatment short of removal by operation at our disposal by which we can undertake to arrest the growth of the tumours, and that the earlier the operation is performed the more easy will it be, Dr. Sharlieb thinks the tendency in the future must be towards early removal, though she recognises the danger that exists of carrying this principle too far.

